# Cofactors As Metabolic Sensors Driving Cell Adaptation in Physiology and Disease

**DOI:** 10.3389/fendo.2017.00304

**Published:** 2017-11-03

**Authors:** Nabil Rabhi, Sarah Anissa Hannou, Philippe Froguel, Jean-Sébastien Annicotte

**Affiliations:** ^1^Lille University, UMR 8199—EGID, Lille, France; ^2^CNRS, UMR 8199, Lille, France; ^3^Institut Pasteur de Lille, Lille, France; ^4^Department of Genomics of Common Disease, School of Public Health, Imperial College London, Hammersmith Hospital, London, United Kingdom

**Keywords:** metabolism, nutritional status, cofactors, epigenetics, metabolites

## Abstract

Chromatin architectures and epigenetic fingerprint regulation are fundamental for genetically determined biological processes. Chemical modifications of the chromatin template sensitize the genome to intracellular metabolism changes to set up diverse functional adaptive states. Accumulated evidence suggests that the action of epigenetic modifiers is sensitive to changes in dietary components and cellular metabolism intermediates, linking nutrition and energy metabolism to gene expression plasticity. Histone posttranslational modifications create a code that acts as a metabolic sensor, translating changes in metabolism into stable gene expression patterns. These observations support the notion that epigenetic reprograming-linked energy input is connected to the etiology of metabolic diseases and cancer. In the present review, we introduce the role of epigenetic cofactors and their relation with nutrient intake and we question the links between epigenetic regulation and the development of metabolic diseases.

## Introduction

During their lifetime, cells receive several external signals, including hormones, growth factors, cytokines and other extracellular factors. Cells translate those signals to make crucial adaptive decisions, such as quiescence, proliferation, or differentiation. Recent works highlighted a fundamental role of environmental cues and nutrient availability in cell metabolism and adaptation. This flow of metabolites, through complex but well characterized metabolic networks, constitutes a fuel for diverse epigenetic cofactors thus relaying nutrition and diet changes into cytoplasmic signaling and chromatin remodeling.

Through their ability to sense internal and external cues, several transcriptional cofactors allow a cell to rapidly adapt by introducing reversible protein posttranslational modifications (PTMs). Hundreds of PTMs have been identified (1–3). However, only few have been directly linked to metabolic fluxes. PTMs include histone and non-histone modifications and represent a key physiological signal for cell adaptation (4–12). For the purposes of this review, we will only focus on PTMs linking changes in metabolism to histone modification.

Histone modifications—all with DNA methylation, RNA interference, and non-coding RNA—encompassed by the term epigenetics represent diverted ways by which cells control the expression of genes without any alteration in the underlying genetic material. Since each cell has the same genetic code, epigenetic modifications allow a fine regulation of the gene expression and determine cell identities. Thereby, various chromatin modification patterns, such as acetylation, methylation, phosphorylation, *O*-linked glycosylation, ubiquitination, and SUMOylation, result in a particular configuration that determines chromatin accessibility to the transcriptional machinery. For example, acetylation of lysine nine residues of histone H3 (H3K9), H3K14, and/or (mono-, di-, or tri) methylation of H3K4, H3K36 and H3K79 are often associated with transcriptionally active chromatin. By contrast, methylation of H3K9, H3K27, and H4K20 are markers of transcriptional silencing (13–15).

The generated metabolites remain the same for a given cell. Yet, the tissue function and nutriment availability will determine the metabolite requirements. Moreover, metabolic challenges, such as caloric or oxygen restriction or even a high-fat diet (16–19), will drive cell fate decisions. Consistent with this, dramatic epigenetic changes have been linked to metabolic disorders, such as obesity, insulin resistance, type-2 diabetes, and cancer (20–25). In this perspective, this review will focus on cofactor families linking nutritional input and metabolism to epigenetic pattern modifications.

## Acetyl-CoA and Histone/Lysine Acetyltransferase Enzymes

Lysine/Histone acetyltransferases (KAT/HAT) catalyze the transfer of an acetyl group from acetyl-CoA to ε-amino group of a histone lysine residue (26), which allows a transcriptional access to DNA by either neutralizing the positive histone charge, or serving as a binding site for chromatin remolding complexes. HAT can be divided on the basis of their subcellular localization or of the structural and functional similarity of their catalytic domains (27).

Acetyl-CoA availability is a major input for histone acetylation. A rise in acetyl-CoA level is sufficient to drive a yeast growth program by promoting histone acetylation at specific growth genes through the General control of amino acid synthesis protein 5-like 2 (GCN5, KAT2A) (28). In mammalian cells, histone acetylation with acetyl-CoA generated from glucose metabolism controls the early differentiation of embryonic stem cells (ESCs) (29). The limiting ATP citrate lyase enzyme that controls the conversion of citrate into oxaloacetate and acetyl-CoA was shown to be important for histone acetylation in response to glucose and growth factor stimulation (30).

As demonstrated for yeast, the mammalian GCN5 activity is required for histone acetylation during cell differentiation (30, 31). Tracing experiments using 13C-carbon combined with acetyl-proteomics showed that up to 90% of histone acetylations on certain histone lysines are derived from fatty acid even in glucose excess. Acetyl-CoA generated from fatty acid β-oxidation seems to be important for the control of a gene expression program involved in lipid metabolism (32). Cytosolic acetate is another acetyl-CoA source that leads to an increase in H3K9, H3K27, and H3K56 histone acetylations of specific promoter regions, enhancing *de novo* lipid synthesis under hypoxic conditions (33). KAT2b is a KAT that acetylates H3K9 and H3K14. During embryogenesis, GCN5 mRNA is already expressed at high levels by day 8, whereas KAT2b mRNA is first detected on day 12.5, suggesting that KAT2b and GCN5 play distinct roles by controlling the expression of a distinct set of genes (34). We have demonstrated that KAT2b is required for pancreatic β-cell adaptation to metabolic stress by promoting histone acetylation and gene expression of several unfolded protein response markers (35). While a β-cell-specific deletion of Kat2b in mouse has no effect under normal diet, Kat2b deficiency leads to a dramatic effect on β-cell morphology and function upon high fat feeding. KAT2b is thereby a major sensor of acetyl-CoA under hyperglycemic condition (35). Altogether, those data suggest that distinct histone acetyltransferases can sense acetyl-CoA upon different conditions and translate the appropriate cell response by activating different sets of genes. Moreover, the origin of acetyl-CoA seems to be important for this selectivity. Sutendra et al. recently demonstrated that acetyl-CoA is generated in the nucleus through a dynamic translocation of the mitochondrial pyruvate dehydrogenase complex (PDC), raising new questions about intracellular acetyl-CoA compartmentalization and the way its origin can regulate a specific set of genes (36–38). A better understanding of KAT activation, of the origin of acetyl-CoA and of its fluctuations within subcellular compartments upon different nutritional challenges can be of interest for the development of new therapeutic strategies against metabolic disease and cancer.

## NAD^+^-Dependent and Independent Histone/Lysine Deacetylases

Lysine/Histone deacetylases (KDAC/HDAC) are the enzymes that catalyze the removal of the acetyl group from lysine residues of histones. On the basis of their mechanistic similarities, they can be divided into two groups: classical HDAC and NAD^+^-dependent sirtuin deacetylase families (39, 40).

The mammalian NAD^+^-dependent KDACs consist of seven sirtuin members (SIRT1 to SIRT7), with distinct subcellular localizations. Three sirtuins are located in the mitochondria (SIRT3–SIRT5), while SIRT1, SIRT6, and SIRT7 are predominantly located in the nucleus, and SIRT2 is found in the cytoplasm (41, 42). NAD^+^ levels rise in energy deficiency situations, such as exercise, caloric restriction, and fasting, leading to sirtuin activation (43, 44). In contrast, when energy is in excess, NAD^+^ is depleted, generating a higher NAD^+^/NADH ratio, which inhibits sirtuin activity (6, 41, 42). This notion further argues toward a direct link between the nutritional status and epigenetic control.

SIRT1, one of the most studied KDAC, controls circadian rhythm and liver metabolism through the deacetylation of H3K9 and H3K14 at the promoter of clock genes (45, 46). Furthermore, through its interaction with Menin, SIRT1 enhances histone deacetylation and controls hepatic triglyceride accumulation (47, 48). SIRT1 can also deacetylate H4K16, functionally linking metabolic activity to genome stability and aging (49, 50). SIRT6, another nuclear sirtuin, is linked to aging by controlling a specific deacetylation of H3K9 at NF-κB target gene promoters (51). In cancer cells, SIRT7 is involved in the stabilization of their transformed phenotype by inducing the deacetylation of H3K18 at specific oncogene promoter regions (52).

The second families of KDAC are classical HDAC, and, in spite of their independent activity on endogenous metabolite, they have been linked to cellular metabolism. Shimazu et al. showed that β-hydroxybutyrate produced under fasting, starvation or intense exercise condition is a natural endogenous HDAC inhibitor leading to increased H3K9 and H3K4 acetylation (18, 53). It also increases histone acetylation at the Foxo3a and Mt2 promoters through the inhibition of HDAC1 and HDAC2 (18).

Lactate production, as a result of an increased rate of glycolysis, has also been shown to upregulate the expression of genes associated with HDAC proteins (54). The authors have demonstrated that the primary effect of lactate on gene expression depends on HDAC inhibition (54). Therefore, lactate may be an important transcriptional regulator, linking the metabolic state of the cell to gene transcription. Further work is needed to corroborate whether the lactate produced *in vivo* has a tissue specific effect on HDAC cofactors. Moreover, lactate has been implicated in the modulation of the DNA damage and repair processes as well as in the resistance of carcinoma cells to anticancer therapy (55).

Altogether, those data provide evidence for a direct link between metabolism products and cellular adaptation through the modulation of KDAC activity.

## Histone Methylation and S-Adenosylmethionine (SAM)

S-adenosylmethionine, generated by the methionine cycle, contains the active methyl donor group used by methyltransferases to methylate RNA, DNA, and proteins, including histones (56–61). While extensive studies focused on the changes of methylation status upon embryonic development, physiology, and diseases, the link between intracellular SAM fluctuation and their conversion into specific epigenetic modifications remains poorly understood. For instance, only histone methylation has been linked to methionine availability, an essential amino acid obtained from the diet (62).

Histone methylation can occur on arginine or lysine residues. While lysine can be mono-, di-, or trimethylated, arginine can only be mono-methylated. There are three classes of histone methyltransferase: SET domain lysine methyltransferases, non-SET domain lysine methyltransferases (disruptor of telomeric silencing 1-like, DOT1L), and arginine methyltransferases (PRMT) (63–65).

In mouse ESCs, mitochondrial threonine dehydrogenase (TDH), an enzyme that catabolizes threonine into glycine and acetyl-CoA, has been shown to be important in maintaining the intracellular SAM level (66). Threonine depletion in culture medium or TDH knockdown in mouse ESCs decreases SAM accumulation and H3K4me3 mark, whereas no effect was observed in other methylation marks (66). In cancer cells, an aberrant expression of Nicotinamide N-methyltransferase—a limiting enzyme that metabolizes SAM—exerts specific control over the cells methylation potential (67). Moreover, recent works provide evidence in both mouse and human that methionine status is sufficient for the control of numerous physiological processes including the activity of genes involved in cell fate through the modulation of histone methylation levels (62).

As observed for HDAC, PMRT activity can be controlled by intermediary metabolites. Three recent reports showed that an increased intracellular concentration of methylthioadenosine (MTA) in cancer cells harboring 5-methylthioadenosine phosphorylase (MTAP) deletions leads to PMRT5 inhibition (68–70). MTAP is the enzyme controlling MTA cleavage to generate precursor substrates for methionine and adenine salvage pathways. In cancer cells, MTAP deficiency leads to partial metabolite-based inhibition of PRMT5 by altering the ratio of MTA to SAM, which results in a decreased H4R3me2s mark (68–70). More studies are needed to understand whether the MTA-to-SAM ratio can also be controlled by physiological metabolic nutritional states.

## Flavin Adenine Dinucleotide (FAD) and Histone Demethylases

Histone methylation was originally considered as a permanent chromatin alteration until the landmark discovery of histone lysine-specific demethylase 1 (LSD1) by Shi Yang’s group, established both *in vitro* and *in vivo* methylation reversibility (71). LSD1 uses FAD formed from ATP and riboflavin (vitamin B2) in mitochondria as a cofactor to demethylate mono- and di-methylated H3K4 and H3K9 (72, 73). Although LSD1 demethylase activity appears to control the metabolism in favor of *de novo* fatty acid synthesis over gluconeogenesis in hepatocyte and brown adipose tissue thermogenic activity, a direct link between nutritional status and LSD1 activity still needs to be established (74–78). For instance, recent works demonstrate that livers from mouse fed with folate-deficient diet present an increased dimethyl-H3K4 and decreased LSD1 activity (79). More studies are needed to decipher the metabolic consequence of FAD fluctuation upon physiological and pathophysiological conditions.

## α-Ketoglutarate (αKG) and Histone Demethylases

α-ketoglutarate is produced from isocitrate through the activity of two key-enzymes of the Krebs cycle, isocitrate dehydrogenase 1 and 2 (IDH 1 and IDH2) (16, 80). αKG can also be produced anaplerotically from glutamate by oxidative deamination, using glutamate dehydrogenase (49). Under fasting or caloric restriction, the accumulation of αKG is used by the αKG-depending dioxygenase to influence the epigenetic status of the cells (81, 82). Several chromatin-modifying enzymes are regulated by αKG availability, including demethylase enzymes containing a Jumonji C domain (JmjC) and ten-eleven translocation (TET) protein families (83–86).

The JmjC subfamily comprises the largest identified family of lysine demethylases (KDMs) with more than 60 enzymes identified in humans (87). In addition to αKG, JmjC-dependent histone demethylation requires iron Fe(II) (88). Each JmjC family member exhibits preference to reverse lysine or arginine trimethylated histone. Considering the key-determinant role of methylation on gene expression and demethylase specificity, KDM2 and KDM5 families have been shown to promote a repression chromatin status, while KDM3, KDM6 and KDM7 act as chromatin activators (63).

Ten-eleven translocation protein family can catalyze 5-methylcytosine (5mC) to 5-hydroxymethylcytosine (5hmC), 5-formylcytosine (5fC) and 5-carboxylcytosine (5caC) in three consecutive Fe(II)- and 2-oxoglutarate (2-OG)-dependent oxidation reactions (89, 90). Gene expression depends on the location of the 5hmC marks. Indeed, the presence of 5hmC in the gene bodies was found to correlate positively with gene expression, whereas no correlation with gene expression was found when 5hmC peaks are located at transcription start sites (91, 92).

α-ketoglutarate can be derived from glucose and glutamine. However, few studies have demonstrated a direct link between αKG generation and histone demethylation. A direct manipulation of intracellular αKG/succinate ratio is sufficient to regulate chromatin state in ESCs. The accumulation of αKG promotes self-renewal of ESCs through JMJD3 and Tet1/Tet2 demethylation of H3K9me3, H3K27me3, and H4K20me histone marks (93). Gas chromatography coupled to mass spectrometry analysis revealed a rapid increase in hepatic α-KG levels following intraperitoneal glucose injection in mice. Strikingly, 5hmC and 5fC marks are reported to increase in various mouse tissues including the liver, kidney, and muscle without any change in TET protein expression or localization leading to a change in gene expression (94). Changes in demethylase activity may thereby contribute to cellular and tissue dysfunction under persistent hyperglycemic conditions.

In cancer cells, a loss of function mutation of TCA cycle enzymes, such as mitochondrial succinate dehydrogenase or fumarate hydratase, promotes succinate and fumarate abundance (95, 96). Both metabolites inhibit α-KG-depending demethylase leading to a decreased 5hmC mark and a specific increase in H3K9me3 levels (96, 97). Somatic mutations of IDH1 and IDH2 have been identified in glioblastomas, acute myelogenous leukemia, chondrosarcomas and lymphomas and other solid tumors (98–103). These gain-of-function mutations lead to a new enzymatic activity promoting the conversion of α-KG to produce D(R)-2-hydroxyglutarate (R2HG) (104, 105). This oncometabolite, which accumulates in tumors with IDH mutations, is a competitive inhibitor of TET and JmjC protein family activity (106–109).

Two recent reports describe another metabolite generated under hypoxic condition by the conversion of α-KG to produce L(S)-2-hydroxyglutarate (S2HG) (110, 111). Both reports demonstrate that S2HG is the product of malate dehydrogenase 1, malate dehydrogenase 2, and lactate dehydrogenase A. The accumulation of this metabolite leads to α-KG-depending demethylase activity inhibition toward TET1/2 and KDM4C (110, 111). Interestingly, this effect is not cancer-specific since a similar level of S2HG production was observed in endothelial cells (110). Moreover, manipulating S2HG is sufficient to increase the methylation of histone repressive marks, suggesting that this metabolite may be generated in other conditions than hypoxia. Further studies are needed to understand the role of S2HG in controlling proliferation versus fate in ES cell.

## Nuclear Localization of Metabolites

The cytosol and nucleus are dense and very viscous. This may restrict the diffusion of small molecules and slow down biochemical reactions. Moreover, several metabolite pathways are organized in multiprotein complexes to allow reaction channeling to facilitate signaling. A multiprotein complex (molecular assembly line) has been proposed to promote efficient substrate channeling from one enzyme to the next (112). Accumulated evidence suggests a close coupling of the histone-modifying enzymes with their critical cofactor synthesis enzyme in the nucleus. Their nuclear translocation aims to provide *in situ* metabolite synthesis in response to metabolic stress. For example, Katoh and colleagues demonstrate that the SAM-generating enzyme, methionine adenosyltransferase II (MATIIα), is localized in the nucleus and interacts with the Swi/Snf and NuRD complexes, supplying SAM for methyltransferases (113). MATIIα will maintain a local high SAM concentration, which is used by an H3K9-specific histone methyltransferase to repress the oncogene MafK transcriptional activity (113).

Similarly, a pyruvate conversion to acetyl-CoA is processed in the nucleus through the nuclear translocation of the mitochondrial PDC. Nuclear PDC levels, as well as the histone H3 and H4 global acetylation levels, increase in a cell-cycle depending manner upon epidermal growth factor, serum, or mitochondrial stress (36). Nuclear PDC inhibition leads to a specific decrease in the acetylation of the histone that is important for the gene expression of G1-S phase progression and S phase markers (36). Moreover, nuclear concentration of acetyl-coA has been shown to be important for osteoblast differentiation (114). In line with this, recent works showed that pyruvate is critical for the TCA cycle enzyme nuclear localization in mammalian zygotic genome activation (115). The authors demonstrated that nutrients, such as pyruvate, are essential for an early pre-implantation development in mouse and human. Mechanistically, Nagaraj and colleagues showed that pyruvate controls the nuclear localization of multiple TCA enzymes in addition to proteins related to TCA cycle entry, including pyruvate carboxylase, pyruvate dehydrogenase, pyruvate dehydrogenase phosphatase, citrate synthase, aconitase-2, and isocitrate dehydrogenase 3A (115). Moreover, acetate-dependent acetyl-coA synthase 2 (ACSS2) binds to chromatin nearby regions of genes that are upregulated during neuron differentiation. A decrease in ACSS2 lowers nuclear acetyl-coA levels, histone acetylation, and neuronal genes in hippocampus, leading to defective spatial memory (116). Those data support a critical role of ACSS2, linking acetate metabolism to localized acetyl-coA production, histone acetylation, and gene expression. In hepatocellular carcinoma cells, the nuclear localization of ACCS2 promotes cancer cell survival by increasing H3K9, H3K27, and H3K56 acetylation levels at the promoter regions of lipogenic genes such as acetyl-CoA carboxylase alpha and fatty acid synthase and enhances *de novo* lipid synthesis (33).

Finally, NAD^+^, the critical cofactor of sirtuin deacetylase, can also be generated in the nucleus through the conversion of nicotinamide by nuclear NMNAT1. NMNAT1 enzymatic activity is required to provide NAD^+^ for SIRT1 (117) and PARP1 (118) during transcriptional regulation and DNA repair.

The precise nuclear localization of critical cofactor-generating enzymes supports the presence of localized subdomains within the chromatin that may promote the clustering of relevant PTMs at specific genomic loci. This model raises a new question on how the nutritional state and metabolism products control the nuclear localization and activity of those microdomains. The second question is to know whether those processes are tissue and cell specific and if they are disturbed under pathophysiological conditions such as obesity or cancer. Then, the final question is: what is the functional and physiological significance of this process?

## Conclusion

Scientific evidence clearly supports that nutrition and diet are the most influential lifestyle factors that contribute to health and the development and progression of chronic diseases, including metabolic disorders, neurodegenerative diseases, cancers, and cardiovascular diseases.

**Figure 1:**
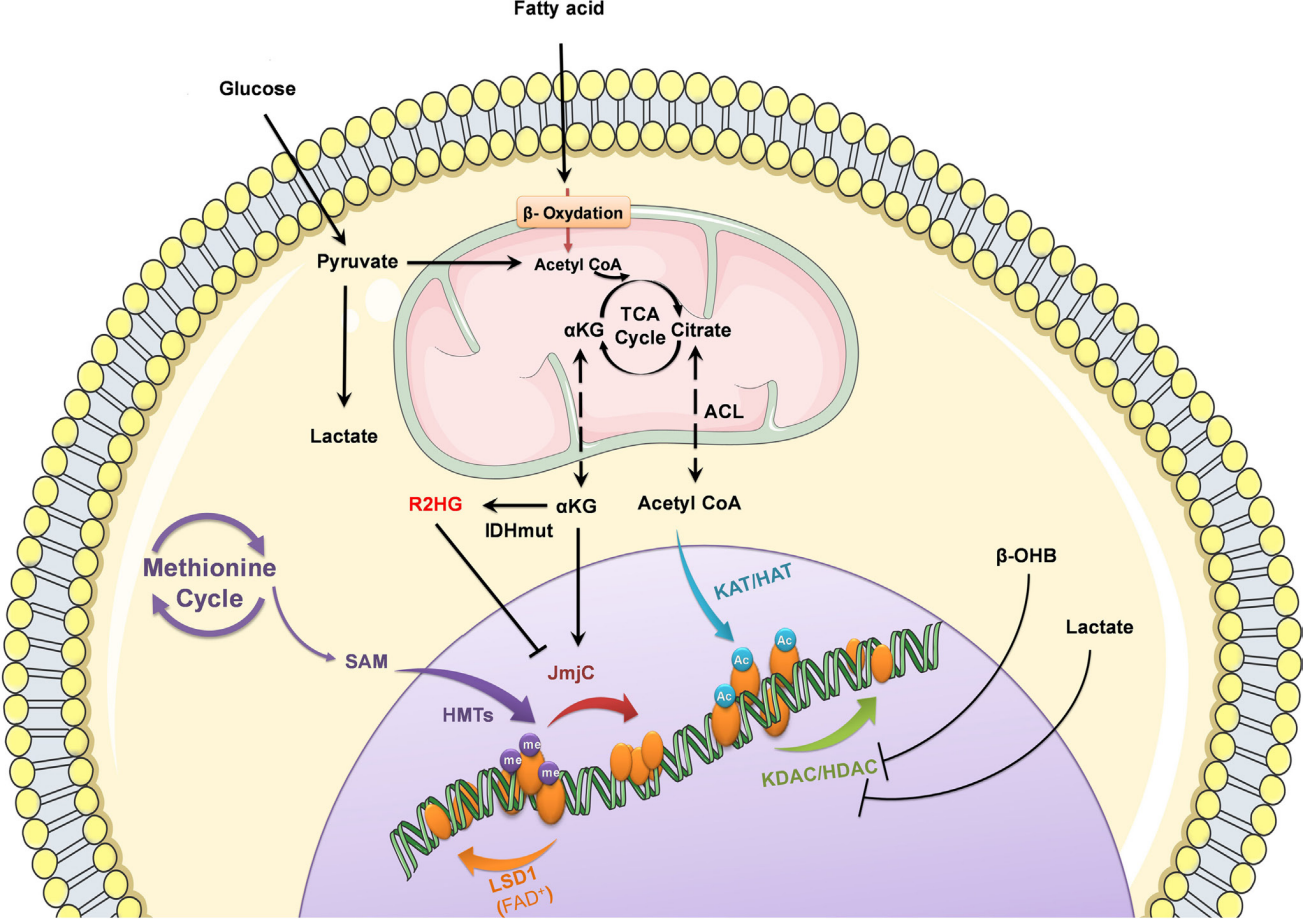
Nutrient-derived metabolites control epigenetic processes. Metabolic status and circulating nutrients, such as glucose and free fatty acids, provide intermediate metabolites required for histone modifications to control gene expression.

The recent exciting advances surveyed herein show that eating habits and nutritional input is deciphered by a metabolic sensor and translated into an adaptive epigenetic code that controls major biological processes such as cell survival, proliferation, DNA damage, and cellular energy production and/or storage (Figure 1). The next major challenge for epigenetic research will depend on the ability to translate the lessons learned from epigenomic profiling, structural studies, and regulatory mechanisms to treatment.

However, it will also be important to strengthen our understanding on how metabolite fluctuations can control a specific gene set in a given tissue. Importantly, it will be of interest to understand how all those pathways integrate into a specific physiological and/or pathophysiological state. The mechanisms controlling the concentration of metabolites in microdomains within the nucleus and the ability for this chromatin compartmentalization of critical cofactor synthesis enzyme to coordinate specific responses to metabolite changes are two other intriguing questions.

Finally, the most important question might be to determine whether cofactors can be successful targets for metabolic diseases. Although this review highlights how far we have come in less than two decades, those findings shed light on a wide range of more open questions to understand the role of cofactors in nutritional sensing and the epigenetic control of gene expression.

## Author Contributions

NR, SAH, J-SA, and PF discussed and wrote the manuscript.

## Conflict of Interest Statement

The authors declare that the research was conducted in the absence of any commercial or financial relationships that could be construed as a potential conflict of interest.
